# Identification of characteristic compounds of moderate volatility in breast cancer cell lines

**DOI:** 10.1371/journal.pone.0235442

**Published:** 2020-06-29

**Authors:** Mitsuru Tanaka, Chung Hsuan, Masataka Oeki, Weilin Shen, Asuka Goda, Yusuke Tahara, Takeshi Onodera, Keisuke Sanematsu, Tomotsugu Rikitake, Eiji Oki, Yuzo Ninomiya, Rintaro Kurebayashi, Hideto Sonoda, Yoshihiko Maehara, Kiyoshi Toko, Toshiro Matsui

**Affiliations:** 1 Department of Bioscience and Biotechnology, Faculty of Agriculture, Kyushu University, Fukuoka, Japan; 2 Research and Development Center for Five-Sense Devices, Kyushu University, Fukuoka, Japan; 3 Faculty of Information Science and Electrical Engineering, Kyushu University, Fukuoka, Japan; 4 Section of Oral Neuroscience, Graduate School of Dental Science, Kyushu University, Fukuoka, Japan; 5 Department of Surgery and Science, Graduate School of Medical Sciences, Kyushu University, Fukuoka, Japan; 6 JOHNAN Co., Kyoto, Japan; 7 Department of General Surgery, Imari-Arita Kyoritsu Hospital, Saga, Japan; 8 Institute for Advanced Study, Kyushu University, Fukuoka, Japan; University of Agriculture in Krakow, POLAND

## Abstract

In this study, we were challenging to identify characteristic compounds in breast cancer cell lines. GC analysis of extracts from the culture media of breast cancer cell lines (MCF-7, SK-BR-3, and YMB-1) using a solid-phase Porapak Q extraction revealed that two compounds of moderate volatility, 1-hexadecanol and 5-(*Z*)-dodecenoic acid, were detected with markedly higher amount than those in the medium of fibroblast cell line (KMST-6). Furthermore, LC-TOF/MS analysis of the extracts clarified that in addition to the above two fatty acids, the amounts of five unsaturated fatty acids [decenoic acid (C10:1), decadienoic acid (C10:2), 5-(*Z*)-dodecenoic acid (C12:1), 5-(*Z*)-tetradecenoic acid (C14:1), and tetradecadienoic acid (C14:2)] in MCF-7 medium were higher than those in medium of KMST-6. Interestingly, H_2_O_2_-oxidation of 5-(*Z*)-dodecenoic acid and 5-(*Z*)-tetradecenoic acid produced volatile aldehydes that were reported as specific volatiles in breath from various cancer patients, such as heptanal, octanal, nonanal, decanal, 2-(*E*)-nonenal, and 2-(*E*)-octenal. Thus, we concluded that these identified compounds over-produced in breast cancer cells in this study could serve as potential precursors producing reported cancer-specific volatiles.

## Introduction

The national epidemiological profiles of cancer burden in the Global Burden of Disease study estimated that there will be 18.1 million new cancer cases and 9.6 million cancer deaths worldwide in 2018; lung cancer is the most commonly diagnosed cancer (11.6% of the total cases), closely followed by breast cancer (11.6%), prostate cancer (7.1%), and colorectal cancer (6.1%) for incidence. [1] The detection and diagnosis of cancer at earlier stages apparently determines further treatments, and periodic health screening using various techniques, such as X-rays, [2] blood tests, [3] mammography, [4] ultrasonography, [4] computed tomography, [5] positron emission tomography, [6] and magnetic resonance imaging, [4] is highly recommended. Regarding definitive diagnosis, an invasive tumour tissue biopsy followed by immunohistochemistry (IHC) of cancer-specific markers, such as oestrogen receptor (ER), progesterone receptor (PR), human epidermal growth factor receptor 2 (HER2), and Ki-67 (a nuclear protein associated with cellular proliferation for breast cancer) is a common technique. [7] However, considering physical stress of patients and the cost for cancer diagnosis, simpler, easier, and faster diagnostic methodologies are still required.

Among the reported cancer-diagnostic methodologies, non-invasive techniques using breath, [8] urine, [9] and hair [10] are approaches that must be of great benefit for initial cancer diagnosis owing to their convenience and rapidity. Our previous report showed that dog could distinguish colorectal cancer patients from non-cancer individuals by smelling their exhaled breath. [11] This strongly suggests the diagnostic benefit of breath as non-invasive technique and presence of cancer-related volatiles in breath, which could be useful for the breath-related research fields. Thus far, a number of volatiles have been identified in exhaled breath or headspace of cell culture media by gas chromatography-mass spectrometry (GC-MS). [12,13] However, it still remains unclear whether volatiles present in breath are specifically derived from cancer disorders, since distinguishing cancer patients’ breath from that of the ones without cancer was achieved by pattern analysis (metabolomics) of volatile compounds, but not by analysis of individual compounds. [14,15] Moreover, it is challenging to reproducibly and precisely determine the volatile levels in exhaled breath due to their low levels (in some cases, below the limit of detection by GC) and environmental contaminations such as ingested food and smoking. [16]

**Fig 1:**
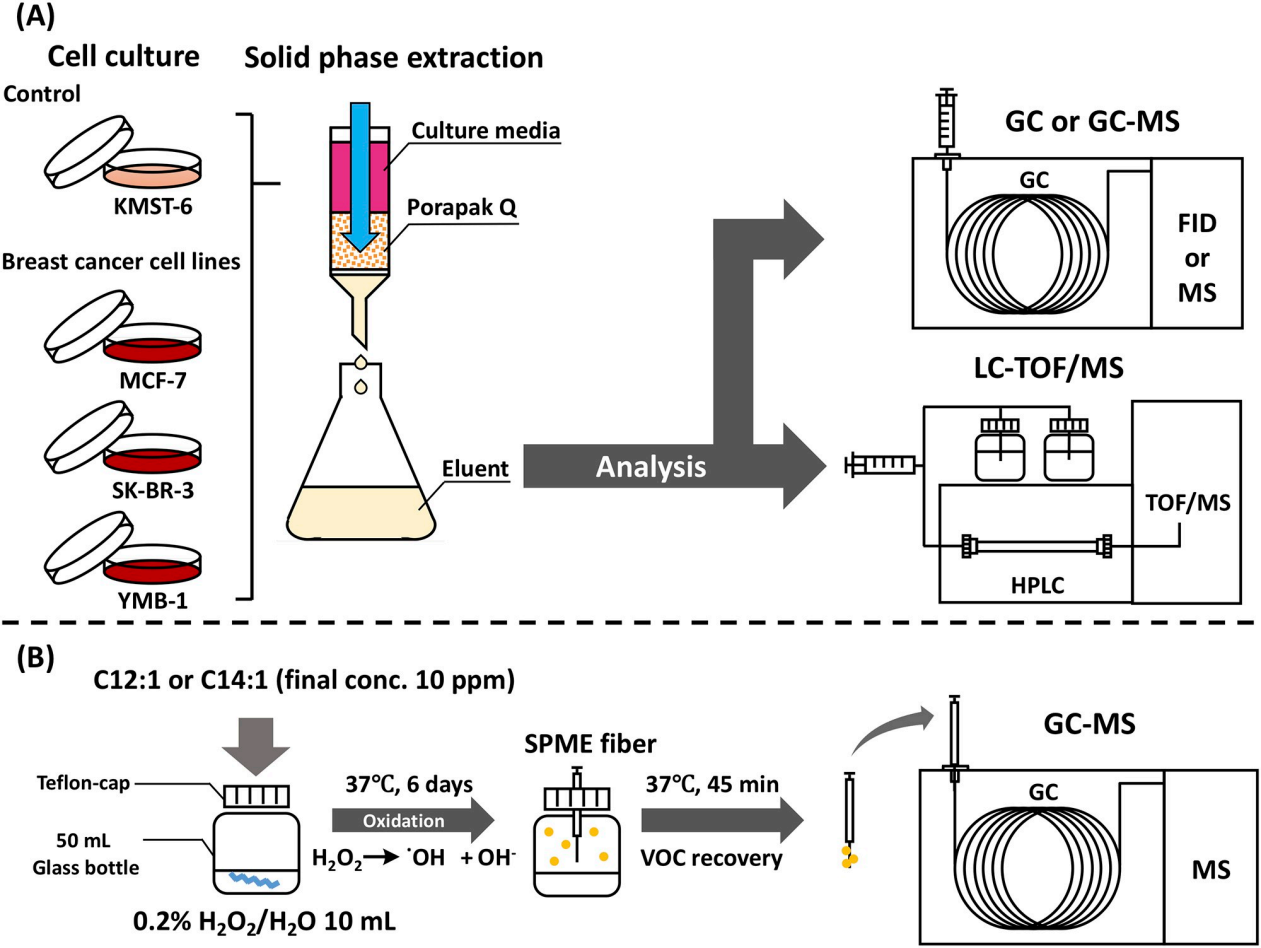
Schematic diagram of analytical protocols for compounds in culture media of breast cancer cell lines by solid-phase Porapak Q extraction (A) and volatiles from H_2_O_2_-oxidation of identified fatty acids by solid-phase micro-extraction (SPME) (B).

Most of the experiments on volatiles in breath from cancer patients so far have been conducted using the solid-phase micro-extraction (SPME) GC-MS method, [12,13] targeting the compounds with high volatility (boiling point: 50 °C–250 °C) [17] in breath or “headspace” of fluids. It is likely that fatty acids and lipids may be precursor candidates responsible for the generation of endogenous volatiles. [18] These findings led us to hypothesis that cancer-specific precursors with low volatility (boiling point: 250 °C–400 °C) may still occur in biological fluids. Thus, since breast cancer is one of the serious problems to be addressed, the aim of this study was to identify such compounds of moderate volatility by targeting breast cancer cells. A Porapak Q (ethylvinylbenzene-divinylbenzene copolymer) resin that is commonly used as GC absorbent was used for the solid-phase extraction of volatiles in the culture media, since the resin displayed an extensive absorption of diverse volatiles with middle to high boiling points. [19,20] GC or GC-MS analysis, in combination with liquid chromatography-time-of-flight (LC-TOF)/MS, were performed to identify compounds that were specifically present in culture media of several breast cancer cell lines (Fig 1A).

## Materials and methods

### Materials and chemicals

5-(*Z*)-Dodecenoic acid (Product number: 445029, Lot number: 09512CR) and 5-(*Z*)-tetradecenoic acid (Product number: T291600, Lot number: 3-WEN-16-3) were purchased from Sigma-Aldrich (St. Louis, MO, USA) and Toronto Research Chemicals (Toronto, Canada), respectively. Linoleic acid (Product number: 2054142, Lot number: M1P5948) was purchased from Nacalai Tesque, Inc. (Kyoto, Japan). 1-Hexadecanol (Product number: H0071, Lot number: X45PJ-MM) was obtained from Tokyo Chemical Industry Co. Ltd. (Tokyo, Japan). All other chemicals were of analytical reagent grade and were used without further purification.

### Cell culture

Human breast cancer cell lines MCF-7 (JCRB0134) and YMB-1 (JCRB0823), and fibroblast cell line KMST-6 (JCRB0433) were obtained from Japanese Collection of Research Bioresources (JCRB Cell Bank, Osaka, Japan). Another human breast cancer cell line SK-BR-3 (No. 30–2007) was obtained from American Type Culture Collection (ATCC, Summit Pharmaceuticals International, Tokyo, Japan). Both JCRB and ATCC provide these cell lines with the confirmation of a negative result of micomycoplasma testing and STR-profiling to characterise the cell lines. Although International Cell Line Authentication Committee (ICLAC) Database of Cross-contaminated or Misidentified Cell Lines reports a risk that YMB-1 is a substrain of breast cancer cell line ZR-75-1, the aim of this study regarding characteristic compounds commonly present in breast cancer cell lines would overcome the issue. MEM media (Wako, Osaka, Japan) for MCF-7 and KMST-6, D-MEM media (Thermo Fisher Scientific, Waltham, MA, USA) for MCF-7, SK-BR-3, and KMST-6, and RPMI 1640 media (Thermo Fisher Scientific) for YMB-1 and KMST-6 were used with supplementation of 10% fetal bovine serum (Sigma). Cells were maintained at 37 °C in humidified atmosphere containing 5% CO_2_. Each cell line was individually seeded and grown in a culture flask (175 cm^2^, IWAKI, Shizuoka, Japan) up to a sub-confluence of 70%–80%. After this growing period, the cells were transferred to a new culture flask (Multi-Flasks 5-layer 875 cm^2^, Corning, NY, USA) at 5.0 × 10^6^ cells/flask. The culture medium of 250 mL/bottle was refreshed every two days and collected into 5L-polypropylene bottle (Steritainer, Sekisui, Osaka, Japan) for 4 days as the medium collecting period (total volume: 500 mL) for solid-phase Porapak Q extraction. Collected culture medium was stored at -40 °C until extraction.

### Extraction of volatile compounds in culture media with solid-phase Porapak Q resin

Volatile organic compounds in culture media of cell lines were extracted using a solid-phase Porapak Q resin according to the procedures described by Fukamachi et al. [21] Medium collected from the cell line culture (500 mL) was injected to a column packed with Porapak Q resin (50–80 mesh, Waters Co., Milford, MA, USA) with a bead volume of 20 mL (50 mm length × 20 mm I.D.). After washing the column with 100 mL of deionized water, diethyl ether (100 mL)-extraction was performed. Contaminated water in the ether eluate was removed by the addition of excess anhydrous sodium sulphate (Nacalai Tesque), followed by the evaporation of ether at 42 °C in a water bath. The obtained ether-concentrate (500 μL) was stored at -30 °C prior to GC or LC analysis.

### GC-FID and GC-MS analyses

Ether concentrate was analysed using a Shimadzu GC-18A equipped with an FID detector (Shimadzu Co., Ltd., Kyoto, Japan) or a Shimadzu GC-MS QP2010 Plus on a DB-FFAP capillary column (30 m × 0.32 mm I.D. with 0.25 μm film thickness, Agilent Technologies, Santa Clara, CA, USA). An aliquot (5 μL) of the concentrate was injected into GC system in splitless mode at a constant carrier gas (He) pressure of 70 kPa with a linear velocity of 40 cm/s. GC separation on the abovementioned capillary column was performed at temperatures programmed at 40 °C for 5 min to 240 °C at 5 °C/min. MS conditions were as follows: electron ionization (EI), positive at 70 eV; mass range, *m/z* 40–500; ion source temperature, 200 °C; interface temperature, 220 °C, and scan event time, 0.3 s. GC-MS identification of target was done by a matching (>90% of similarity) mass spectra with the NIST 14 library database, as well as matching of retention index (RI) [22] of target with that of the standard.

### LC-TOF/MS analysis

Ether concentrate was 10-fold diluted with methanol prior to LC-TOF/MS analysis. LC separation was performed using an Agilent 1200 series HPLC (Agilent Technologies) on a Cosmosil 5C_18_-MS-II column (2.0 mm × 150 mm, Nacalai Tesque) under a linear gradient of 0.1% formic acid (FA) to 100% methanol containing 0.1% FA over 30 min at 0.2 mL/min and 40 °C. MS analysis in a single negative electrospray-ionization (ESI) mode was performed using a micrOTOF-II mass spectrometer (Bruker Daltonics, Bremen, Germany). ESI-MS conditions were as follows: drying N_2_ gas flow rate, 8.0 L/min; drying temperature, 200 °C; nebulizer gas pressure, 1.6 bar; capillary voltage, 3800 V. At the beginning of each run, *m/z* was calibrated by 10 mM sodium formate. Identification of targets was achieved by matching of mass unit and retention time on LC column with standards. Data acquisition and analysis were carried out using a Bruker Data Analysis 3.2 software.

### H_2_O_2_-oxidation of unsaturated fatty acids and identification of volatiles by SPME-GC-MS

H_2_O_2_ oxidation of 5-(*Z*)-dodecenoic acid and 5-(*Z*)-tetradecenoic acid was performed as follows (Fig 1B); each unsaturated fatty acid (10 ppm) was added to 10 mL of 0.2% H_2_O_2_ solution in a teflon-capped 50 mL-vial and the vial stood for 6 days at 37 °C. A blank experiment in 0.2% H_2_O_2_ solution without fatty acid was also performed at the above-mentioned incubation conditions. Extraction of volatile compounds in the headspace gas of the teflon-capped 50 mL-vial was performed with SPME fibre coated with 50/30 μm divinylbenzene/carboxen/polydimethylsiloxane (DVB/CAR/PDMS, SUPELCO Co., Bellefonte, PA, USA). The fibre was conditioned at 270 °C for 1 h. After the vial was placed in a water bath at 37 °C for 20 min, the SPME fibre was exposed to the headspace of the vial for 45 min. The fibre was, then, transferred to an injection port of GC-MS at 250 °C for 10 min for desorption of SPME-absorbed compounds. GC-MS was conducted with a Shimadzu GC-MS QP2010 Plus on a DB-WAX capillary column (30 m × 0.32 mm I.D. with 0.5 μm film thickness, Agilent Technologies). Chromatographic and MS conditions were the same as aforementioned. GC-MS identification of targets was done by matching (>90% of similarity) of mass spectra with the NIST 14 library database, as well as matching of RI of targets with that of the standards.

#### Statistical analyses

The statistical significance between two groups was analysed using unpaired two-tailed Student’s *t*-test. All analyses were conducted with a GraphPad Prism 5 software (GraphPad Software, La Jolla, CA, USA).

## Results

### Identification of characteristic compounds present in culture media of breast cancer cells by GC and GC-MS

**Fig 2:**
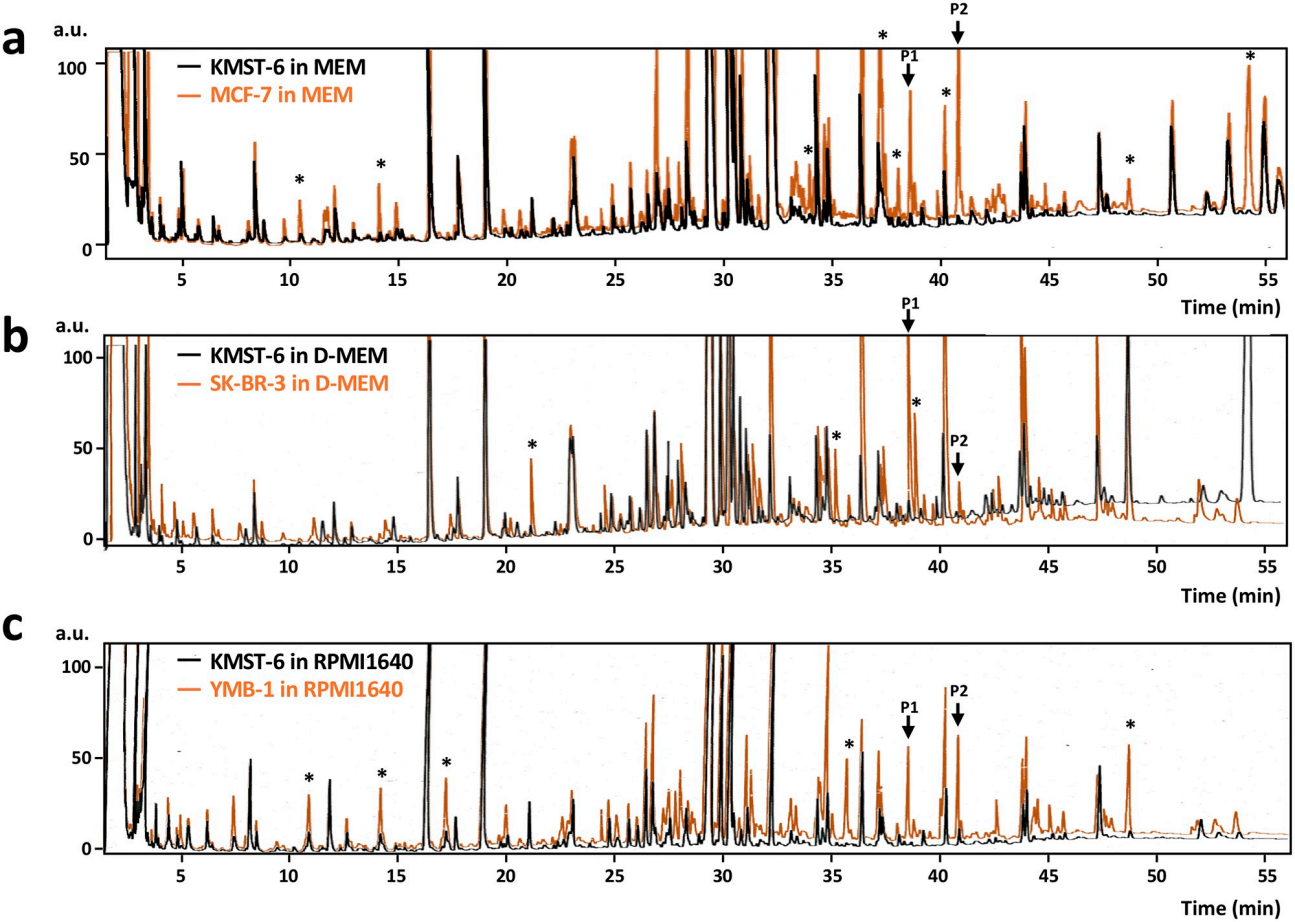
Comparison between GC-FID chromatograms of culture media for breast cancer cell lines [MCF-7 (a), SK-BR-3 (b), and YMB-1(c)] and those for fibroblast cell (KMST-6).

For GC and GC-MS analyses of volatiles in the culture media, three breast cancer cell lines, MCF-7, SK-BR-3, and YMB-1, were cultured in MEM, D-MEM, and RPMI 1640 media, respectively. The culture media were collected at every 2 days for 4 days (total volume of 500 mL) and were subjected to solid-phase extraction using a Porapak Q resin. A fibroblast cell line, KMST-6, as a control cell was cultured in the aforementioned three media respectively and each was subjected to solid-phase extraction. Concentrated ether-extract (500 μL) from solid-phase Porapak Q resin was applied to the gas chromatograph with a flame-ionization detector (GC-FID) to identify the peaks specific for breast cancer cell lines, since FID can widely detect organic compounds without any consideration of detection efficiency (as in the case of MS ionization) between compounds. On comparing GC chromatograms of KMST-6 media with those of breast cancer cell media, we observed enhanced GC peaks that were common in all three different cell lines. However, enhanced peaks that did not commonly appear in the three different media (marked with asterisk in Fig 2) were excluded from the possibility of being breast cancer-specific volatile candidates; e.g., peaks at 10.8 min, 14.1 min, 33.8 min, 37.2 min, 38.0 min, 40.1 min, 48.6 min, and 54.1 min for MCF-7 (Fig 2a), peaks at 21.2 min, 35.8 min, and 38.9 min for SK-BR-3 (Fig 2b), and peaks at 10.8 min, 14.2 min, 17.2 min, 35.7 min, and 48.6 min for YMB-1 (Fig 2c). According to the report by Hanai et al., [23] two peaks (denoted as P1 and P2 in Fig 2) showing significantly and commonly high intensity in the three different breast cancer cell media were selected (Fig 2a–2c); GC peak intensities of P1 and P2 in cell cultures of MCF-7 were > 6-times higher than those in KMST-6 (*n* = 3, *P* < 0.05) (S1 Fig). In addition, P1 and P2 observed in MEM medium for MCF-7 (Fig 2a) were also detected when MCF-7 was cultured in D-MEM (S2 Fig). This indicated that the two peaks were generated from cancer cell growth, but not medium composition.

GC-FID samples from 500 mL of culture media (MEM for MCF-7, D-MEM for SK-BR-3, RPMI 1640 for YMB-1) were obtained through solid-phase Porapak Q extraction. KMST-6 cells were also present in each culture medium for comparison of their GC chromatograms as controls, with those of cancer cell media. Analytical conditions for solid-phase Porapak Q extraction and GC-FID analysis on DB-FFAP capillary column (30 m × 0.32 mm I.D.) were described in the Methods section. GC chromatograms obtained from the extracts of breast cancer cells and KMST-6 cells were in orange and black colour respectively. Asterisk (*) indicates the predominant peaks for cancer cells within the same media of cancer and control KMST-6 cells. Peaks denoted as P1 and P2 are predominant peaks commonly observed in all cancer cell extracts regardless of the media.

**Fig 3:**
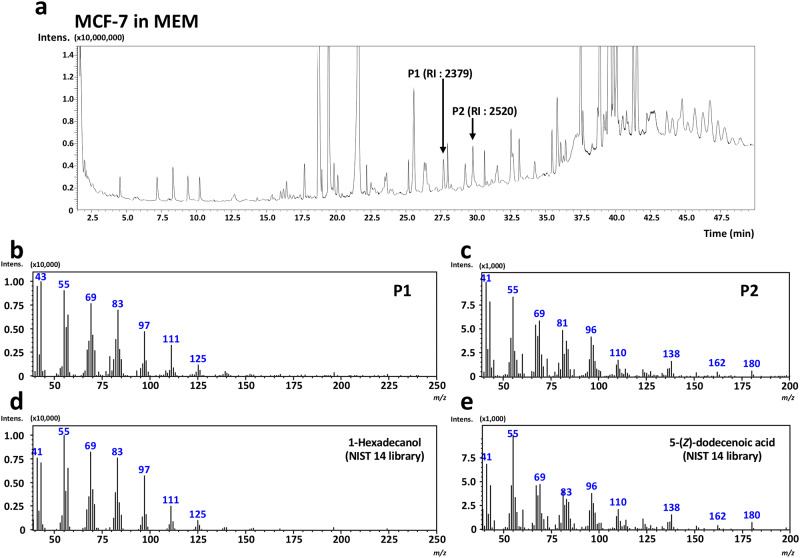
GC-MS identification of 1-hexadecanol and 5-(*Z*)-dodecenoic acid in culture media of MCF-7 breast cancer cells. Culture media (MEM) of MCF-7 breast cancer cells were subjected to solid-phase Porapak Q extraction. The extracts were injected to GC-MS. Targeted peaks selected by GC-FID analyses (i.e., P1 and P2) on GC-MS total ion chromatogram (a) were determined according to RI values of 2379 and 2520, respectively. MS spectra of P1 (b) and P2 (c) were applied to similarity search with NIST 14 library database, and a similarity score of 93% for 1-hexadecanol (d) and 95% for 5-(*Z*)-dodecenoic acid (e) was obtained, respectively. Analytical conditions for GC-MS on DB-FFAP capillary column (30 m × 0.32 mm I.D. with 0.25 μm film thickness) were described in the Methods section.

Identification of P1 and P2 was performed using GC-MS with the aid of a similarity search using NIST library 14 software. As shown in Fig 3, P1 and P2 were identified as 1-hexadecanol (NIST similarity score of 93) and 5-(*Z*)-dodecenoic acid (NIST similarity score of 95), respectively. The two compounds of moderate volatility were also confirmed by their standards [1-hexadecanol: RI on FFAP column, 2379; 5-(*Z*)-dodecenoic acid: RI on FFAP column, 2520] (S3 Fig). To the best of our knowledge, this is the first finding showing that the two compounds obtained through GC analysis of the culture media were possible candidates for breast cancer cell lines.

### LC-TOF/MS analysis of 5-(*Z*)-dodecenoic acid and the related compounds specific for MCF-7 breast cancer cell culture medium

**Fig 4:**
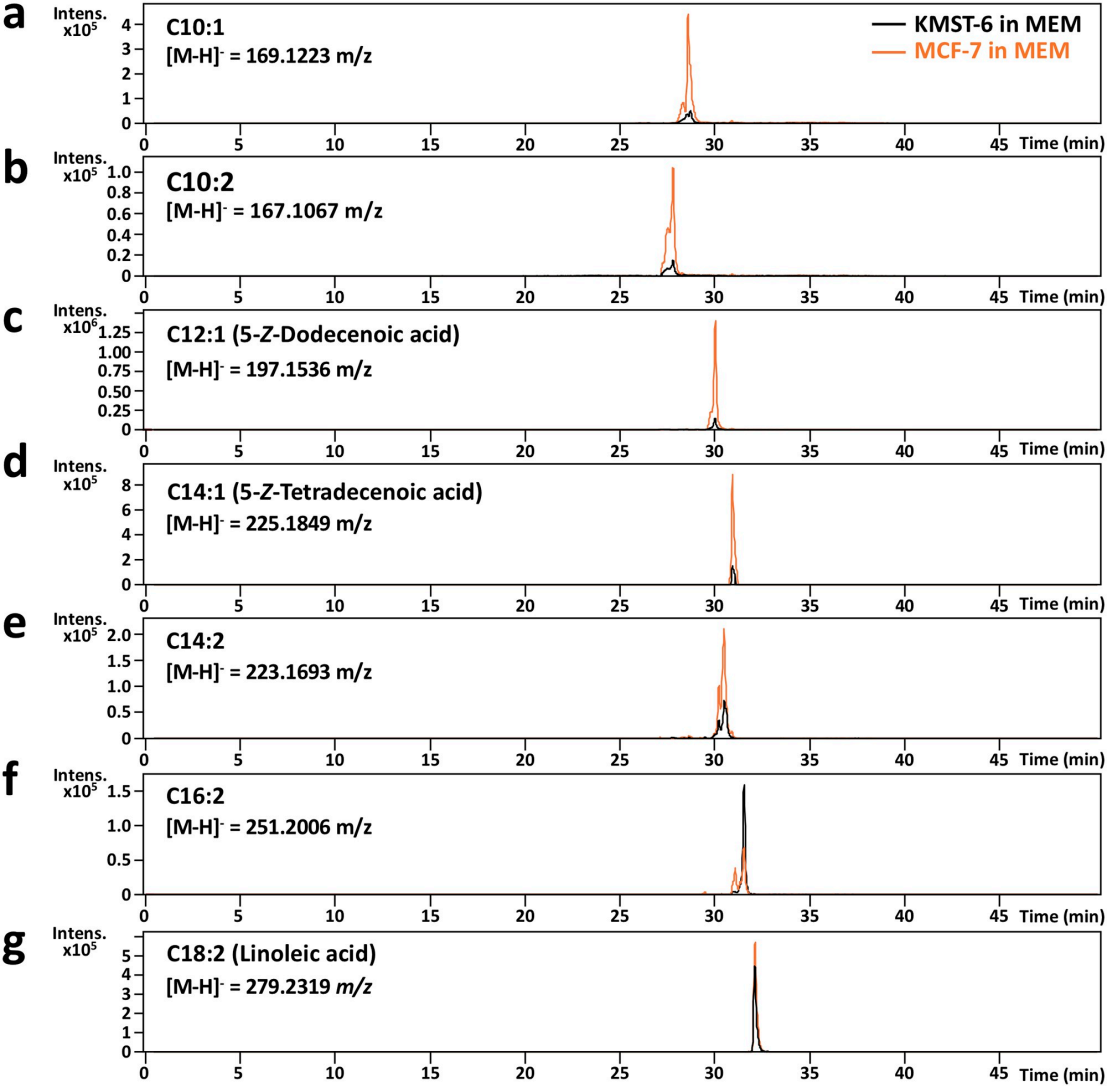
LC-TOF/MS identification of 5-(*Z*)-dodecenoic acid and its related-unsaturated fatty acids in culture media of MCF-7 breast cancer cells. Culture media of MCF-7 breast cancer cells were subjected to solid-phase Porapak Q extraction. Ten-fold diluted extracts with methanol were injected to LC-TOF/MS. Targeted *m/z* values for 5-(*Z*)-dodecenoic acid and related-unsaturated fatty acids for EIC-LC-TOF/MS analysis were as follows: (a) C10:1 ([M-H]^-^ = 169.1223 *m/z*), (b) C10:2 ([M-H]^-^ = 167.1067 m/z), (c) C12:1 [5-(*Z*)-dodecenoic acid: [M-H]^-^ = 197.1536 *m/z*], (d) C14:1 [5-(*Z*)-tetradecenoic acid: [M-H]^-^ = 225.1849 *m/z*], (e) C14:2 ([M-H]^-^ = 223.1693 *m/z*), (f) C16:2 ([M-H]^-^ = 251.2006 m/z), and (g) C18:2 (linoleic acid: [M-H]^-^ = 279.2319 *m/z*). EIC-MS chromatograms obtained from the extracts of breast cancer MCF-7 and fibroblast KMST-6 cells were in orange and black colour, respectively. Analytical conditions for LC-TOF/MS on a Cosmosil 5C_18_-MS-II column (2.0 mm × 150 mm) were described in the Methods section.

Considering that 5-(*Z*)-dodecenoic acid could be a possible biomarker for breast cancer, further experiments were performed to investigate whether 5-(*Z*)-dodecenoic acid-related unsaturated fatty acids were also abundant in MCF-7 cell culture media compared to those in KMST-6. LC-TOF/MS (but not GC-MS) analysis of MCF-7 cultured MEM medium was performed owing to aqueous properties of the targeted fatty acids in medium. As shown in Fig 4a–4g, seven peaks on extracted-ion chromatograms (EIC) corresponding to *m/z* values of unsaturated fatty acids (C10:1, C10:2, C12:1, C14:1, C14:2, C16:2, and C18:2) were detected, and five peaks (C10:1, C10:2, C12:1, C14:1, and C14:2) showed much higher intensity in MCF-7 compared to those in KMST-6 (Fig 4a–4e). With the aid of commercially available standards, C12:1 and C14:1 detected in MCF-7 were successfully identified as 5-(*Z*)-dodecenoic acid and 5-(*Z*)-tetradecenoic acid, respectively, whereas the position of the unsaturated bond for other unsaturated fatty acids (except for C18:2 as linoleic acid) could not be defined due to unavailability of standards.

### Generation of volatiles from 5-(*Z*)-dodecenoic and 5-(*Z*)-tetradecenoic acids by H_2_O_2_-oxidation

**Fig 5:**
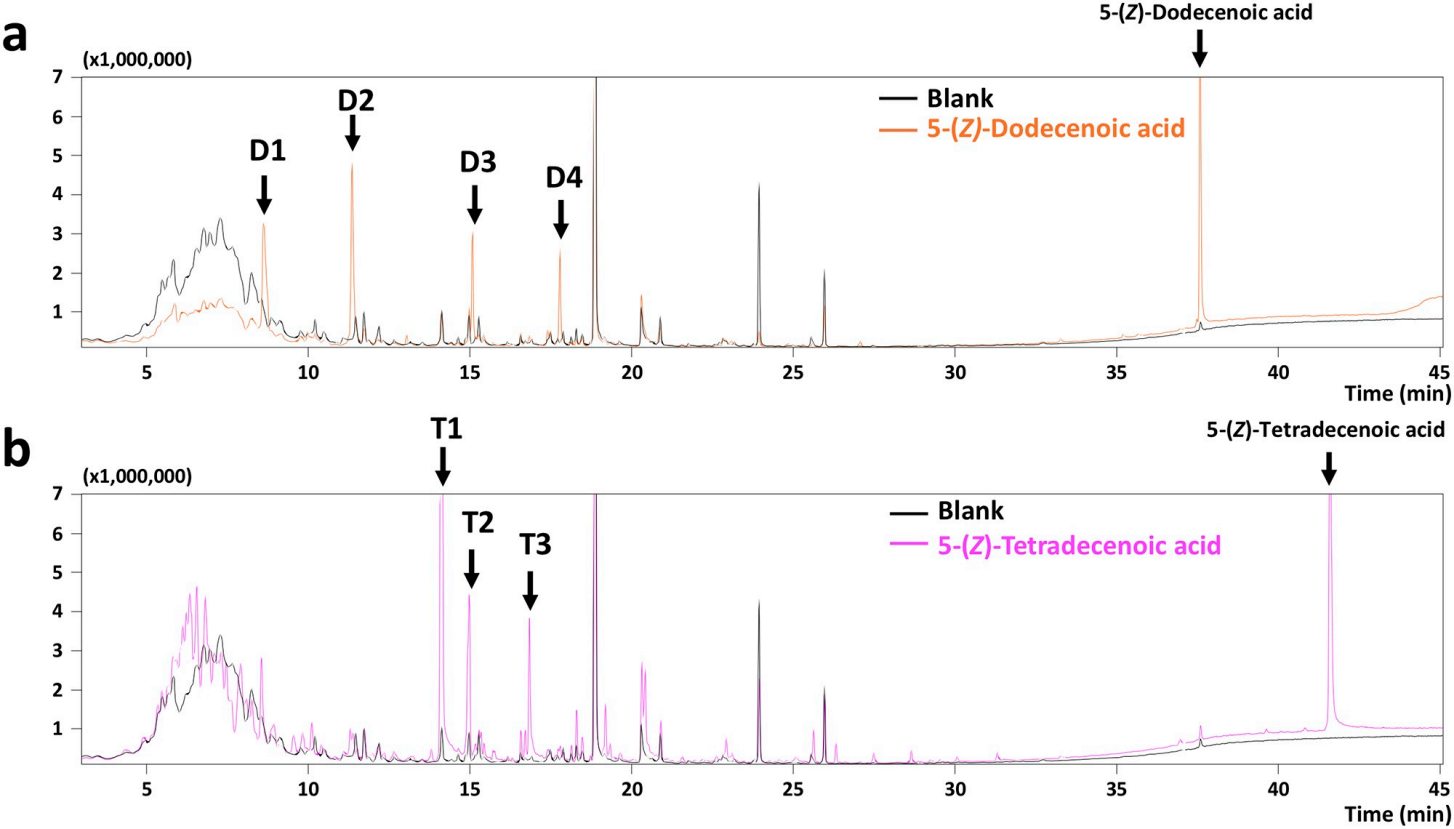
GC-MS analyses of volatiles generated from 5-(*Z*)-dodecenoic acid (a) and 5-(*Z*)-tetradecenoic acid (b) by H_2_O_2_-oxidation. H_2_O_2_ (0.2%) oxidation of 5-(*Z*)-dodecenoic acid and 5-(*Z*)-tetradecenoic acid (each 10 ppm in deionized water) was performed respectively in a 50-mL teflon-sealed vial at 37 °C for 6 days. A blank experiment in 0.2% H_2_O_2_ solution without fatty acid was also performed at the above-mentioned incubation conditions. Volatiles in the headspace of the vial were analysed by SPME-aided GC-MS analysis on a DB-WAX capillary column (30 m × 0.32 mm I.D.). Analytical conditions for SPME extraction and for GC-MS analysis on DB-FFAP capillary column (30 m × 0.32 mm I.D.) were described in the Methods section. Newly observed peaks with high MS intensity were denoted as D1 to D4 and T1 to T3 for 5-(*Z*)-dodecenoic acid (a) and 5-(*Z*)-tetradecenoic acid (b), respectively.

Since unsaturated fatty acids that are liable to oxidation may cause the production of unexpected volatiles, [24] further experiments were performed to identify the unsaturated fatty acid-derived volatiles by 0.2% H_2_O_2_-accelerating oxidation of each acid in water (10 ppm) in a 50 mL-sealed vial for 6 days at 37 °C. As shown in Fig 5, GC-FID analysis of the 6-day-incubates of 5-(*Z*)-dodecenoic acid and 5-(*Z*)-tetradecenoic acid revealed that four peaks from 5-(*Z*)-dodecenoic acid and three peaks from 5-(*Z*)-tetradecenoic acid were newly and markedly obtained compared to GC chromatograms of the blank (without both acids) sample. GC-MS analysis in combination with RI matching [22] on DB-WAX column showed that all the identified compounds except T2 were volatile aldehydes (Tables 1 and 2). In addition, these identified aldehydes (heptanal, octanal, nonanal, decanal, 2-(*E*)-nonenal, and 2-(*E*)-octenal) were volatiles identified in the breath or blood from patients of various cancers as reported previously, [8,25–29] e.g., heptanal in breath as a lung cancer-specific volatile. [8,25,26] This finding allowed us to speculate that the unsaturated fatty acids identified in this study may be one of the possible precursors of the reported cancer-specific volatiles.

**Table 1:** Identified volatiles from 5-(*Z*)-dodecenoic acid by H_2_O_2_-oxidation.

Peak	Rt (min)	RI on DB-WAX	Compound	Reported identification
**D1**	**8.63**	**1192**	**heptanal**	**breath for lung cancer** [8,25]
				**breath for breast cancer** [27]
				**plasma for bone cancer** [29]
				**plasma for Burkitt’s lymphoma** [29]
				**plasma for large cell lymphoma** [29]
**D2**	**11.35**	**1296**	**octanal**	**breath for lung cancer** [8,26]
**D3**	**15.08**	**1437**	**2-(*E*)-octenal**	**plasma for bone cancer** [29]
				**plasma for Burkitt’s lymphoma** [29]
				**plasma for large cell lymphoma** [29]
**D4**	**17.78**	**1544**	**2-(*E*)-nonenal**	**plasma for acute myelogenous leukemia** [29]

**Table 2:** Identified volatiles from 5-(*Z*)-tetradecenoic acid by H_2_O_2_-oxidation.

Peak	Rt (min)	RI on DB-WAX	Compound	Reported identification
**T1**	**14.14**	**1400**	**nonanal**	**breath for lung cancer** [8,26]
				**breath for ovarian cancer** [28]
				**breath for colorectal cancer** [30]
**T2**	**14.97**	**1433**	***N*.*I***.^a^	**-**
**T3**	**16.83**	**1505**	**decanal**	**breath for ovarian cancer** [28]
				**breath for colorectal cancer** [30]

^a^*N*.*I*., not identified.

## Discussion

So far, research interests have been focused on the elucidation of “volatiles” in the exhaled breath of cancer patients, since the evidential report that dog could distinguish colorectal cancer patients by smelling their exhaled breath. [11] Thus, research on volatiles in breath from cancer patients [8,12] have been conducted using SPME extraction method, which is suitable for the recovery of highly volatile compounds from gas phase. In contrast, in the present study, we used a solid-phase Porapak Q resin for the recovery of a variety of volatiles, since compounds of moderate volatility such as fatty acids or lipids were targeted as precursors of these cancer specific volatiles. For the first time, we identified two compounds, 1-hexadecanol and 5-(*Z*)-dodecenoic acid, by GC-FID analysis in the culture media of MCF-7, a breast cancer cell line (Fig 2a). Both newly identified compounds were observed in breast cancer cell media regardless of the species of the breast cancer cell line (MCF-7, SK-BR-3, and YMB-1, Fig 2a–2c) and medium (MEM, D-MEM, and RPMI 1640, S2 Fig).

Considering the boiling points of 344 °C and 311 °C for 1-hexadecanol and 5-(*Z*)-dodecenoic acid, respectively, they could evaporate into gas phase, since compounds with a high boiling point of > 300 °C, e.g., β-naphthyl methylketone with boiling point of 301 °C, can behave as a synthetic strawberry-like flavour compound. [24] Thus, a direct analysis of the headspace of culture media of breast cancer cells is needed for further experiments, which is now in progress to confirm their vaporization into gas phase. Abaffy et al. reported that 1-hexadecanol was identified as one of the malignant melanoma-specific volatiles in melanoma biopsy. [31] Shigeyama et al. also revealed that 1-hexadecanol was newly produced in the saliva of oral squamous cell carcinoma patients, together with other alcohols and ketones as volatile metabolites. [32] Therefore, it seems likely that 1-hexadecanol was a candidate cancer-specific biomarker.

It has been revealed that BRCA1 germline mutation, and HER2 and ER expressions were candidate tumour biomarkers for the risk of breast or ovarian cancer, and for therapeutic assessment of breast and gastric cancers, respectively. [33] Serum biomarkers such as carcinoembryonic antigen (CEA), cancer antigen 19–9 (CA19-9), cancer antigen 125 (CA125), cancer antigen 15–3 (CA15-3), and tissue polypeptide-specific antigen (TPS) have also been used as diagnostic metabolites for metastatic breast cancer. [3] In contrast, challenging studies on non-invasive cancer diagnosis have been reported using breath, [8] urine, [9] or hair. [10] Although the non-invasive approach appears to be of great value owing to convenient and rapid sampling, the reliability of diagnostic results remains unascertained. For example, although many candidate volatiles in exhaled breath of breast cancer subjects have been reported so far, [12] no crucial volatiles that can characterise the onset of breast cancer have been elucidated yet. In this study, we successfully identified two unsaturated fatty acids, 5-(*Z*)-dodecenoic acid and 5-(*Z*)-tetradecenoic acid, in the culture media of breast cancer cells. It is well known in food sciences that unsaturated fatty acids such as oleic, linoleic, and linolenic acids are liable to oxidation, causing the generation of volatile aldehydes and ketones responsible for “fatty and green” odour quality. [24] In this study, autoxidation treatment of 5-(*Z*)-dodecenoic acid and 5-(*Z*)-tetradecenoic acid by H_2_O_2_ caused the production of volatile aldehydes including heptanal, octanal, nonanal, decanal, 2-(*E*)-nonenal, and 2-(*E*)-octenal (Fig 5a and 5b, and Tables 1 and 2) in agreement with the reported cancer-specific volatiles. [8,25–29] Although the production behaviour of volatiles from the two fatty acids was obtained in the present limited H_2_O_2_-oxidation experiments, the identified unsaturated fatty acids in the culture media of breast cancer cells may behave as a precursor for the generation of cancer-specific volatiles seen in exhaled breath of lung, [8,25,26] breast, [27] ovarian, [28] and colorectal cancer patients, [30] as well as plasma from cancer patients who are children. [29]

Cancer can alter fatty acid metabolism in response to hypoxia inducible factor-1 (HIF-1)-mediated attenuation of medium chain acyl-CoA dehydrogenase (MCAD) and long chain acyl-CoA dehydrogenase (LCAD). [34] It has been reported that MCAD or LCAD deficiency caused the increase in medium chain fatty acids in human plasma, such as C8:0, C10:1, and C10:2 for MCAD deficiency, and C12:1 [5-(*Z*)-dodecenoic acid], C14:1 [5-(*Z*)-tetradecenoic acid], C14:2 and C16:2 for LCAD deficiency. [35] In the MEM culture medium of MCF-7, 5 unsaturated fatty acids (C10:1, C10:2, C12:1 [5-(*Z*)-dodecenocic acid], C14:1 [5-(*Z*)-tetradecenoic acid], and C14:2) were successfully detected with a > 2.5-fold higher intensity than those in KMST-6 (Fig 4a–4e). Thus, according to the present observations, the increase of medium chain unsaturated fatty acid identified in this study in plasma or other biological fluids would be a detectable symptom for breast cancer onset. We are now investigating diagnostic potential of target fatty acids as biomarkers for breast cancer using non-invasive biological fluids.

In conclusion, in this study, we firstly demonstrated that 1-hexadecanol and 5-(*Z*)-dodecenoic acid (and its related unsaturated fatty acids including 5-(*Z*)-tetradecenoic acid) were important candidates in breast cancer cells but not fibroblast cells by GC-MS and/or LC-MS analyses of cell culture media. We also found that unsaturated fatty acids could generate the reported volatiles specific for breath from various cancer patients such as heptanal, octanal, nonanal, decanal, 2-(*E*)-nonenal, and 2-(*E*)-octenal by H_2_O_2_ oxidation. Thus, the diagnostic targets, medium chain unsaturated fatty acids of moderate volatility, would overcome the issues on poor reproducibility and precision of volatile detection in exhaled breath. [16] In summary, the present findings clearly indicate that unsaturated fatty acids of moderate volatility may be over-produced in breast cancer cells, serving as potential precursors for the production of cancer-specific volatiles reported so far.

## Supporting information

S1 Fig GC-FID intensities of P1 (1-hexadecanol) and P2 (5-(*Z*)-dodecenoic acid) in cell culture media of KMST-6 and MCF-7. GC-FID samples from 500 mL of MCF-7 and KMST-6 culture media (MEM) were obtained through solid-phase Porapak Q extraction. Analytical conditions for solid-phase Porapak Q extraction and for GC-FID analysis on a DB-FFAP capillary column (30 m × 0.32 mm I.D.) were described in the Methods section. GC peak intensities from individual triplicate cell cultures are shown as mean ± S.E.M. Significant differences between groups were analysed by unpaired two-tailed Student’s *t*-test. Significantly high GC intensities of P1 and P2 were obtained in cell culture of MCF-7 compared to KMST-6 (*n* = 3, *P* < 0.05).
(TIFF)

S2 Fig Comparison between GC-FID chromatograms of D-MEM culture medium for breast cancer MCF-7 and those for fibroblast cell (KMST-6). GC-FID samples from 500 mL of MCF-7 culture media (D-MEM) were obtained through solid-phase Porapak Q extraction. KMST-6 cells were also present in the same culture medium for comparison of their GC chromatograms as controls, with those of cancer cell media. Analytical conditions for Porapak Q column extraction and for GC-FID analysis on a DB-FFAP capillary column (30 m × 0.32 mm I.D.) were described in the Methods section. GC chromatograms obtained from the extracts of MCF-7 and KMST-6 were in orange and black colour, respectively. Peaks denoted as P1 and P2 are predominant commonly observed in all cancer cell extracts regardless of different media (as shown in Fig 2).
(TIFF)

S3 Fig GC-FID chromatograms of RPMI 1640 culture medium for breast cancer YMB-1 spiked with 1-hexadecanol (a) or 5-(*Z*)-dodecenoic acid (b). Standard 1-hexadecanol (a) or 5-(*Z*)-dodecenoic acid (b) (1 ppm for each standard) was spiked into Porapak Q-column extract of RPMI 1640 culture medium for YMB-1 to be subjected for GC-FID analysis. Analytical conditions for Porapak Q column extraction and for GC-FID analysis on a DB-FFAP capillary column (30 m × 0.32 mm I.D.) were described in the Methods section. GC-FID chromatograms obtained from standard spiked-media and those not from standard spiked-media were in orange and black colour, respectively.
(TIFF)
