# Supplementary figures and images for: Identification of characteristic compounds of moderate volatility in breast cancer cell lines
Source: PLoS One. 2020 Jun 29;15(6):e0235442. doi: 10.1371/journal.pone.0235442 (PMC7323966; doi:10.1371/journal.pone.0235442)

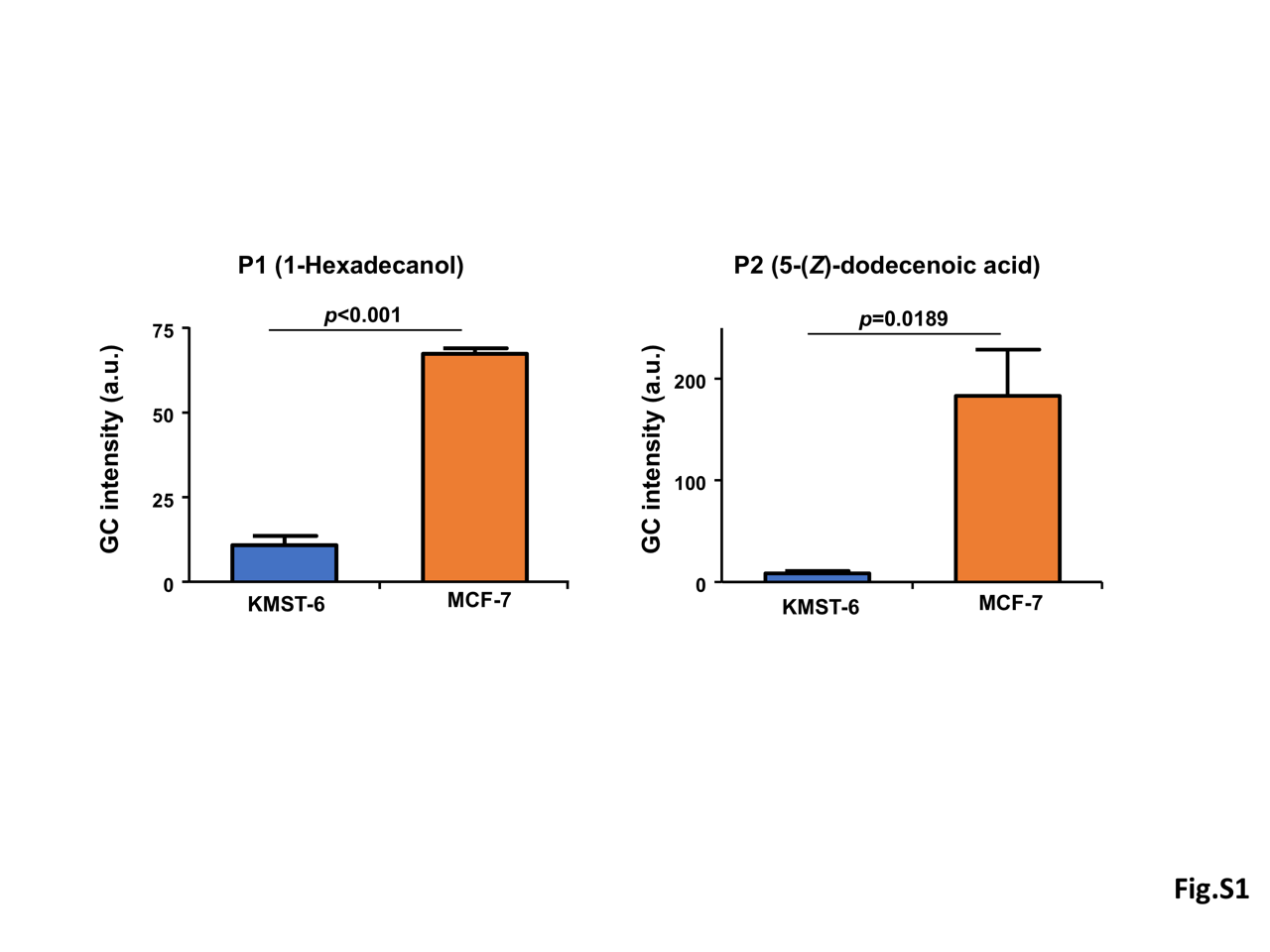

Supplement: S1 Fig — GC-FID samples from 500 mL of MCF-7 and KMST-6 culture media (MEM) were obtained through solid-phase Porapak Q extraction. Analytical conditions for solid-phase Porapak Q extraction and for GC-FID analysis on a DB-FFAP capillary column (30 m × 0.32 mm I.D.) were described in the Methods section. GC peak intensities from individual triplicate cell cultures are shown as mean ± S.E.M. Significant differences between groups were analysed by unpaired two-tailed Student’s t-test. Significantly high GC intensities of P1 and P2 were obtained in cell culture of MCF-7 compared to KMST-6 (n = 3, P < 0.05). (TIFF) [file pone.0235442.s001.tiff]

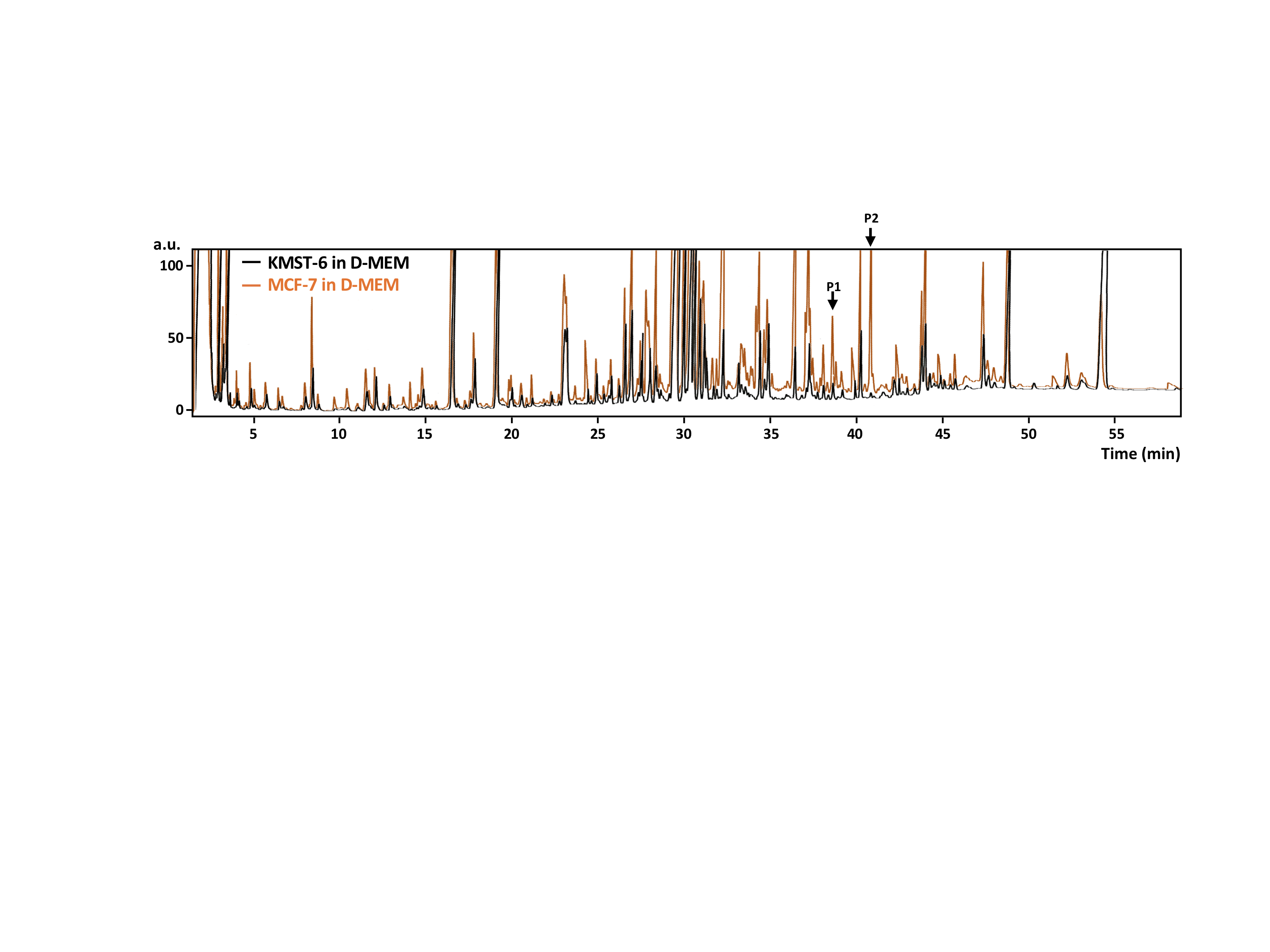

Supplement: S2 Fig — GC-FID samples from 500 mL of MCF-7 culture media (D-MEM) were obtained through solid-phase Porapak Q extraction. KMST-6 cells were also present in the same culture medium for comparison of their GC chromatograms as controls, with those of cancer cell media. Analytical conditions for Porapak Q column extraction and for GC-FID analysis on a DB-FFAP capillary column (30 m × 0.32 mm I.D.) were described in the Methods section. GC chromatograms obtained from the extracts of MCF-7 and KMST-6 were in orange and black colour, respectively. Peaks denoted as P1 and P2 are predominant commonly observed in all cancer cell extracts regardless of different media (as shown in Fig 2). (TIFF) [file pone.0235442.s002.tiff]

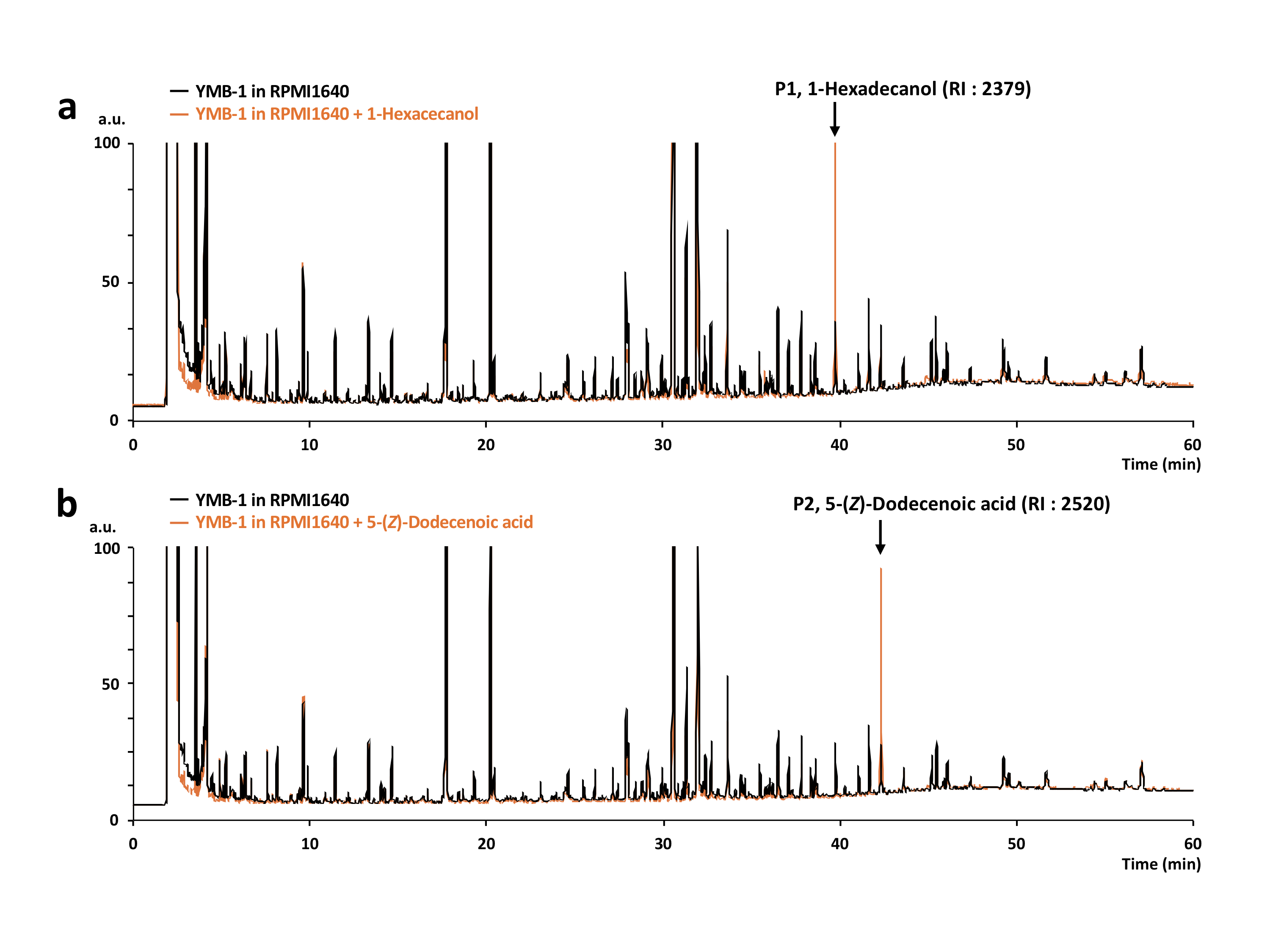

Supplement: S3 Fig — Standard 1-hexadecanol (a) or 5-(Z)-dodecenoic acid (b) (1 ppm for each standard) was spiked into Porapak Q-column extract of RPMI 1640 culture medium for YMB-1 to be subjected for GC-FID analysis. Analytical conditions for Porapak Q column extraction and for GC-FID analysis on a DB-FFAP capillary column (30 m × 0.32 mm I.D.) were described in the Methods section. GC-FID chromatograms obtained from standard spiked-media and those not from standard spiked-media were in orange and black colour, respectively. (TIFF) [file pone.0235442.s003.tiff]
